# Vitamin C Activates the Folate-Mediated One-Carbon Cycle in C2C12 Myoblasts

**DOI:** 10.3390/antiox9030217

**Published:** 2020-03-05

**Authors:** Armando Alcazar Magana, Ralph L. Reed, Rony Koluda, Cristobal L. Miranda, Claudia S. Maier, Jan F. Stevens

**Affiliations:** 1 Department of Chemistry, Oregon State University, 153 Gilbert Hall, Corvallis, OR 97331, USA; alcazara@oregonstate.edu (A.A.M.); koludar@oregonstate.edu (R.K.); claudia.maier@oregonstate.edu (C.S.M.); 2 Linus Pauling Institute, Oregon State University, 2900 SW Campus way, Corvallis, OR 97331, USA; reedr@oregonstate.edu (R.L.R.); cristobal.miranda@oregonstate.edu (C.L.M.); 3 Department of Pharmaceutical Sciences, Oregon State University, 1601 SW Jefferson Way, Corvallis, OR 97331, USA

**Keywords:** vitamin C, folic acid, one-carbon metabolism, C2C12 cells, metabolomics, mass spectrometry, ascorbic acid

## Abstract

Vitamin C (L-ascorbic acid, AA) is an essential cellular antioxidant and cofactor for several α-ketoglutarate-dependent dioxygenases. As an antioxidant, AA interacts with vitamin E to control oxidative stress. While several reports suggest an interaction of AA with folate (vitamin B9) in animals and humans, little is known about the nature of the interaction and the underlying molecular mechanisms at the cellular level. We used an untargeted metabolomics approach to study the impact of AA on the metabolome of C2C12 myoblast cells. Compared to untreated cells, treatment of C2C12 cells with AA at 100 µM resulted in enhanced concentrations of folic acid (2.5-fold) and 5-methyl-tetrahydrofolate (5-methyl-THF, 10-fold increase) whereas the relative concentrations of 10-formyl-tetrahydrofolate decreased by >90% upon AA pretreatment, indicative of increased utilization for the biosynthesis of active THF metabolites. The impact of AA on the folate-mediated one-carbon cycle further manifested itself as an increase in the levels of methionine, whose formation from homocysteine is 5-methyl-THF dependent, and an increase in thymidine, whose formation from deoxyuridine monophosphate (dUMP) is dependent on 5,10-methylene-THF. These findings shed new light on the interaction of AA with the folate-mediated one-carbon cycle and partially explain clinical findings that AA supplementation enhances erythrocyte folate status and that it may decrease serum levels of homocysteine, which is considered as a biomarker of cardiovascular disease risk.

## 1. Introduction

Vitamin C (ascorbic acid, AA) has important functions in animals and humans to maintain health and prevent disease [1]. AA acts as a cofactor for several α-ketoglutarate-dependent dioxygenases, such as prolyl hydroxylase involved in collagen synthesis [2]. Its capacity to donate electrons gives AA antioxidant properties [1] and ability to scavenge electrophiles in Michael additions [3,4]. The antioxidant properties of AA can manifest in many ways and affect a multitude of metabolic pathways. For example, AA supplementation in humans lowers the production and urinary excretion of lipid peroxidation-derived reactive aldehydes [5]. From metabolomics analyses of zebrafish fed a diet lacking sufficient AA, we established that AA deficiency, which causes oxidative stress, activates the purine nucleotide cycle to regenerate ATP [6]. Recently, we reported that AA prevents cellular nitrate tolerance in glyceryl trinitate-treated porcine renal epithelial cells, which is relevant to angina [7]. Using a mass spectrometry-based metabolomics approach, we found that AA protects xanthine oxidase from glyceryl trinitrate-induced inactivation, which is relevant because this enzyme plays a role in the conversion of glyceryl trinitrate into the vasoactive metabolite, nitric oxide [7]. These examples illustrate how a data-driven metabolomics approach can reveal novel functions of AA. 

AA is well known to interact with vitamin E in cellular redox cycles to control oxidative stress [2] and prevent oxidative stress damage to proteins and DNA [8]. Interactions between AA and folic acid (vitamin B9) have also been reported [9,10], although the underlying mechanism remains poorly understood. Folic acid plays a key role as a one-carbon carrier molecule in methylation reactions—notably, the conversion of homocysteine (Hcy) into methionine and the conversion of deoxyuridine monophosphate (dUMP) into deoxythymidine monophosphate (dTMP) [11]. Given that elevated plasma Hcy is considered a risk factor for cardiovascular disease [12] and given the reports of an AA–folate interaction [9,10], AA supplementation has been explored as an attractive way to increase circulating levels of folic acid and to reduce Hcy levels, with mixed results [10,13], although the clinical significance of the interaction and the underlying molecular mechanism remain poorly understood.

Humans cannot synthesize AA and depend on dietary AA for their survival. Most non-primate animals produce AA in the liver [14]. Thus, human and most mammalian cell lines grown in culture do not synthesize AA. Unless cultured cells are supplemented with AA, they are less protected against oxidative stress than their in vivo counterparts. To elucidate the biochemical pathways modulated by AA supplementation, we examined the changes in the metabolome of mouse C2C12 cells in response to AA supplementation. We selected these cells because they are metabolically active and susceptible to oxidative stress [15]. Using an unbiased, untargeted mass spectrometry-based metabolomics approach, we measured major shifts in pool sizes of tetrahydrofolate (THF) metabolites and of substrates of methylation reactions, which prompted us to focus our investigation on the effects of AA supplementation on the folate-mediated one-carbon cycle and associated pathways. Because metabolomics measures relative levels of individual metabolites, the technique provides clues as to which pathways and which enzymatic steps in those pathways are modulated by AA treatment. Here we report that AA facilitates the reductive steps in the conversion of 10-formyl-THF into 5-methyl-THF, which provides new insights in the interaction between AA and folic acid and how the interaction promotes methylation reactions to provide substrates for amino acid and DNA synthesis. 

## 2. Materials and Methods 

### 2.1. Reagents

LC–MS-grade methanol and water were purchased from EMD Millipore (Burlington, MA, USA). Formic acid ACS reagent was from Fisher Chemicals (Suwanee, GA, USA). ACS reagents folic acid, folinic acid (5-formyltetrahydrofolic acid), 5-methyltetrahydrofolic acid, methionine-(methyl-d_3_) (used as internal standard) and butylated hydroxytoluene were purchased from Sigma Aldrich (St. Louis, MO, USA). Sodium ascorbate was from Merck. ^13^C_6_-ascorbic acid was from Omicron Biochemicals, Inc. (South Bend, IN, USA). DMEM was from Life Technologies (Grand Island, NY, USA). Penicillin, streptomycin and fetal bovine serum were from Invitrogen (Carlsbad, CA, United States).

### 2.2. Cell Culture and Treatment

C2C12 cells were obtained from ATCC (Manassas, VA, USA) and were first propagated in 75 cm^2^ flasks using a culture medium consisting of DMEM, 10% fetal bovine serum, 100 units/mL penicillin, and 100 μg/mL streptomycin. The cells were harvested from the flask using trypsin and seeded in 10 cm culture dishes containing 10 mL of the supplemented DMEM medium, which contains 14 amino acids, including methionine, serine, and glycine, and nine vitamins including folic acid but no vitamin C. Immediately after seeding, the cells were treated with 100 µM sodium ascorbate or 100 µM ^13^C_6_-AA by adding 10 µL of the respective 100 mM stock solution. The rationale behind using the two isotopologues of AA was that this approach offered us a tool to assign mass spectral features to AA or its metabolites in the metabolomics dataset. At this concentration, AA supplementation did not change the pH of the medium. Control cells were grown in the absence of AA. All three groups (biological triplicates) were maintained under sterile conditions with 5% CO_2_ at 37 °C. After 48 h of incubation, cells were scraped, counted using a Hemacytometer Counting Chamber Bright-line Neubauer Cell counter (Hausser Scientific, Horsham, PA, USA) according to the manufacturer’s instructions, and spun down at 4 °C at 500× *g* for 5 min. The pellets (equivalent to 3.5 × 10^6^ cells per dish) were washed three times with cold PBS and transferred to 1.7 mL Eppendorf tubes. The pellets were frozen and stored at –80 °C until sample preparation for LC–MS/MS analysis. 

### 2.3. LC–MS/MS Analysis

Cells were resuspended in 300 µL of ice-cold 50:50 (*v:v*) methanol:ethanol containing 0.02% *w/v* of butylated hydroxytoluene (to prevent post-harvest oxidation) and disrupted by sonication for 3 × 15 s in a Fisher Scientific sonifier (1/2 inch disruptor horn, 4 kHz) on ice with 30 s between steps. Samples were spun for 10 min at 14,000× *g* at 4 °C and the supernatant was transferred to mass spectrometry vials (Microsolv, Leland, NC, USA). LC–MS/MS was carried out with a method previously described [6,7,15] with some differences. Briefly, data-dependent acquisition (DDA) in both the positive and the negative ion mode was conducted using a Shimadzu Nexera UPLC system connected to an AB SCIEX TripleTOF^®^ 5600 mass spectrometer (AB SCIEX, Concord, Canada). Samples were randomized before injections. A quality control (QC) sample, obtained by mixing equal aliquots of all samples, was analyzed every five LC runs. The injection volume was 5 μL. Chromatographic separation was performed using an Inertsil Phenyl-3 column (4.6 × 150 mm, 100 Å, 5 µm; GL Sciences, Rolling Hills Estates, CA, USA). A gradient with two mobile phases (A, water with 0.1% *v/v* formic acid and B, methanol with 0.1% *v/v* formic acid) was used. The elution gradient was as follows: 0 min, 5% B; 1 min, 5% B; 11 min, 30% B; 20 min, 100% B; 25 min, 100% B; 30 min, 5% B; and 35 min, 5% B. Period cycle time was 950 ms; accumulation time 100 ms; *m/z* scan range 100–1200; and collision energy 35 V with collision energy spread (CES) of 15 V. Mass calibration was automatically performed after every fifth LC run.

### 2.4. Metabolomics Data Processing

Raw data processing was performed using Progenesis QI^TM^ software with Metlin^TM^ plugin V1.0.6499.51447 (NonLinear Dynamics, United Kingdom) for peak picking, retention time correction, peak alignment, data normalization and metabolite annotations. Annotation confidence was achieved in accordance with reporting criteria for chemical analysis suggested by the Metabolomics Standards Initiative (MSI) [16,17]. Thus, level 1 (L1) annotations were made based on accurate mass (error < 10 ppm), fragment ion spectral pattern (library score > 50), isotope pattern (library score > 80), and retention time (error < 5%) comparison of analytes with those of authentic standards in our in-house library (> 700) analyzed under identical conditions.

For metabolites not present in our in-house library, level 2 (L2, tentative) annotations were made according to the MSI guidelines [16,17] and based on accurate mass, fragment ion spectral pattern, and isotope pattern comparison with online data available in METLIN (which has MS/MS experimental data) and the Human Metabolome Database (which contains in silico generated MS/MS data). Ions generated from QC samples were retained for annotation and included in the dataset if the coefficient of variation (CV) of their abundance did not exceed 25% (QC distribution is shown in supplementary Figure S1). When metabolites were detected in both ion modes, the one with the highest signal-to-noise ratio for a particular metabolite was kept. Relative quantities of metabolites were determined by calculating their corresponding peak areas. 

### 2.5. Statistical Analysis

The area under the curve of molecular ions was selected for relative quantitation of annotated metabolites. Only annotated metabolites were used for statistical analysis. Figures (principal component analysis, heatmaps, dendrograms and biplots) were generated with MetaboAnalyst 4.0 (Montreal, Quebec, Canada) [18] and PowerPoint 2016 (Microsoft, Redmond, WA, USA). Plots were generated with GraphPad Prism 8.3.0 (La Jolla, CA, United States). Metabolite changes were tested by a one-way analysis of variance followed by Tukey’s HSD (honestly significant difference) post-hoc analysis and Holm FDR (false discovery rate) correction, with a *p*-value of < 0.05 indicating significance. Unless otherwise stated, data are presented as the mean ± SD of three independent experiments (*n* = 3/group). In the tables, the two vitamin C treatment groups were collapsed into a single AA group (*n* = 6). To compensate for unequal variance or non-normal distribution, data were logarithmically transformed and Pareto scaled (mean centered and divided by the square root of the standard deviation). No outliers were excluded from the statistical analyses. 

## 3. Results

Metabolomics analysis was performed on mouse skeletal muscle-derived C2C12 myoblasts that were AA deficient or AA supplemented. We selected C2C12 cells because we have experience with metabolome measurements of this cell line, they are metabolically active, and because they are susceptible to oxidative stress [15]. We exposed the cells to two isotopologue forms of AA, sodium ascorbate and ^13^C_6_-ascorbic acid, because the approach allowed us to distinguish between exposure compounds (AA and its metabolites or degradation products) and the effects of the exposure on the metabolome. We did not find evidence that exogenous AA supplementation interfered with annotation of endogenous metabolites. We also did not detect AA in the non-supplemented cells, which is expected because mice produce AA in the liver and not in muscle tissue (Figure S2).

**Figure 1:**
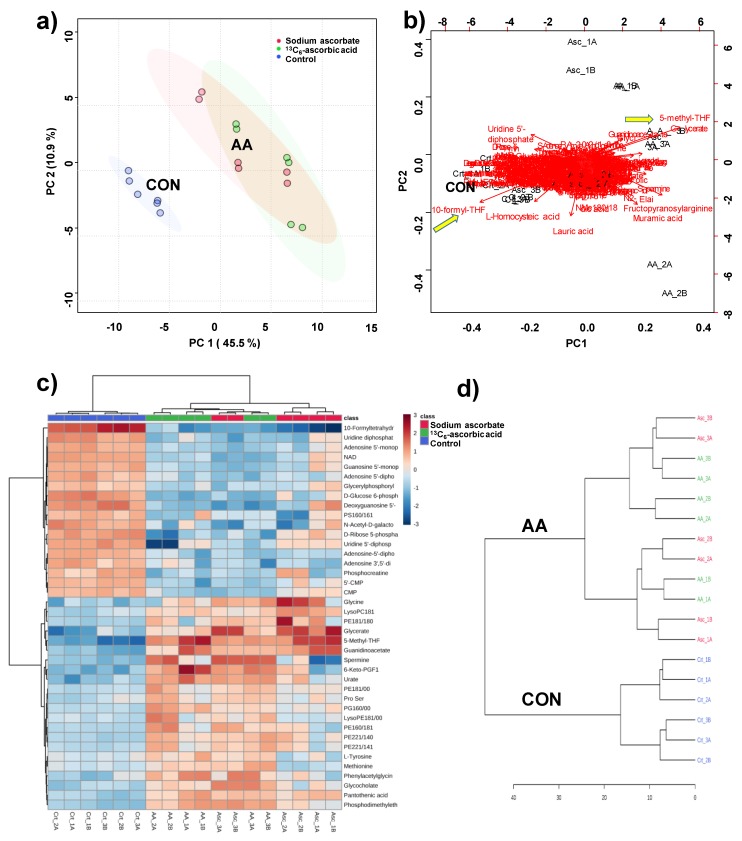
Analysis of treated C2C12 cells with ascorbic acid and with ascorbate 100 µM. (**a**) Principal component analysis (PCA) scores plot, where each dot represents a sample analysis (thus, 6 dots per experiment resulting from biological triplicates and technical duplicates); (**b**) PCA biplot visualizes which metabolites contribute most to the separation of the experimental groups; (**c**) heatmap visualizing the top 40 most differentiating metabolites. Color coding indicates greater deviation from the mean of all samples for a particular metabolite; (**d**) dendrogram indicating the degree of similarity among samples, constructed on the basis of all 204 metabolites. The analysis was performed using MetaboAnalyst V4.0.

The identity of 102 metabolites was assigned using our in-house library (L1 metabolites, supplementary Table S1), while an additional 102 metabolites were assigned by comparison with spectral data available from METLIN and the HMDB (L2 metabolites, supplementary Table S1). The resulting 204 metabolites were further investigated to determine how and to what extent AA exposure altered the cellular metabolome. Figure 1 shows an analysis of similarities and dissimilarities among the treatments. Principal component analysis (PCA) grouped together supplemented cells and separated them from AA-deficient cells (Figure 1a), which demonstrates that the cells responded similarly to exposure to either sodium ascorbate or ^13^C_6_-ascorbic acid. 

A PCA biplot, a representation that shows an overlay of the score and loading plots, visualizes the changing metabolome in response to AA supplementation (Figure 1b). It revealed that the following metabolites were most prominently associated with the group separation of the AA-deficient (controls) from the AA-supplemented cells in the scatter plot of the PCA (Figure 1a): folic acid, 10-formyl-tetrahydrofolate (10-formyl-THF), 5-methyl-THF, homocysteic acid (oxidized form of homocysteine, Hcy), and uridine-5´-diphosphate (UDP) (Figure 1b). A heatmap of the top 40 most differentiating metabolites also shows that the AA-supplemented cells responded differently from the control group (Figure 1c). Using the variation in all 204 annotated metabolites, a similar dendrogram representation emerged (Figure 1d), in which the non-supplemented cells clustered together as did the AA-supplemented cells without distinction between sodium ascorbate- and ^13^C_6_-ascorbic acid-treated cells.

**Figure 2:**
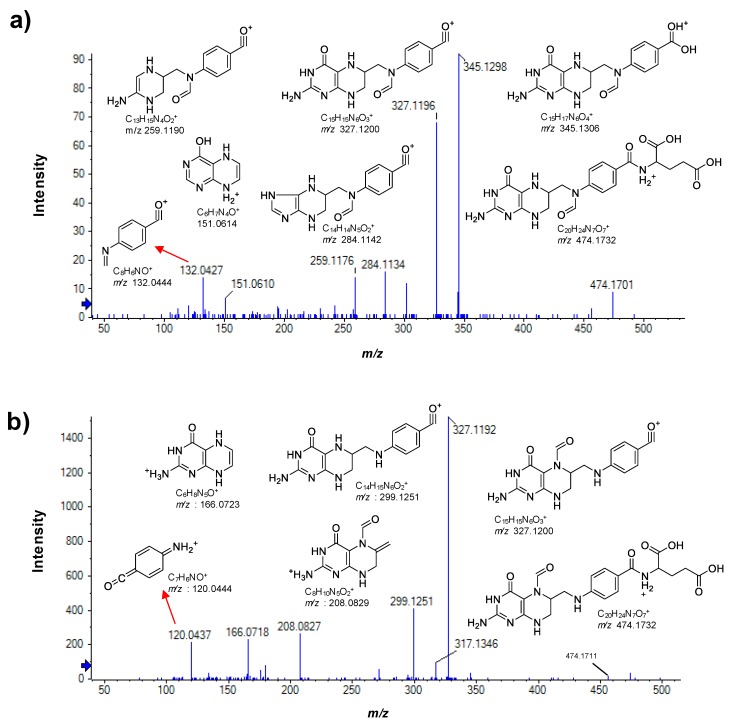
Positive ion mode MS/MS spectra of the two detected formyl-tetrahydrofolate (THF) isomers and the proposed structures of their fragment ions (CE 35 V; CES of 15 V). (**a**) 10-formyl-tetrahydrofolate (10-formyl-THF) and (**b**) 5-formyl-tetrahydrofolate (5-formyl-THF, authentic standard). Peak labels denote accurate masses and fragment ion *m/z* values denote exact masses.

Of the three folic acid metabolites we detected, the formyl derivative of THF was not identical with an authentic standard of 5-formyl-THF with regard to retention time and MS/MS fragmentation pattern (Figure 2). Comparison of the spectra of both formyl derivatives revealed that 5-formyl-THF produced a low-mass fragment with *m/z* 120.0437 which was absent in the spectrum of the regioisomer shown in Figure 2a. Instead, the corresponding fragment of the regioisomer appeared at *m/z* 132.0427. We identified the *m/z* 120.0437 fragment of 5-formyl-THF as ^+^H_2_N=(C_6_H_4_)=C=O and the *m/z* 132.0427 fragment ion of the regioisomer as CH_2_=N–(C_6_H_4_)–C≡O^+^, the difference corresponding to an imino substituent on the nitrogen atom. As this nitrogen atom corresponds to atom position 10 in the precursor ion and considering that the imino group is formed upon loss of a H_2_O molecule from the formyl substituent, we can identify the regioisomer as 10-formyl-THF.

**Figure 3:**
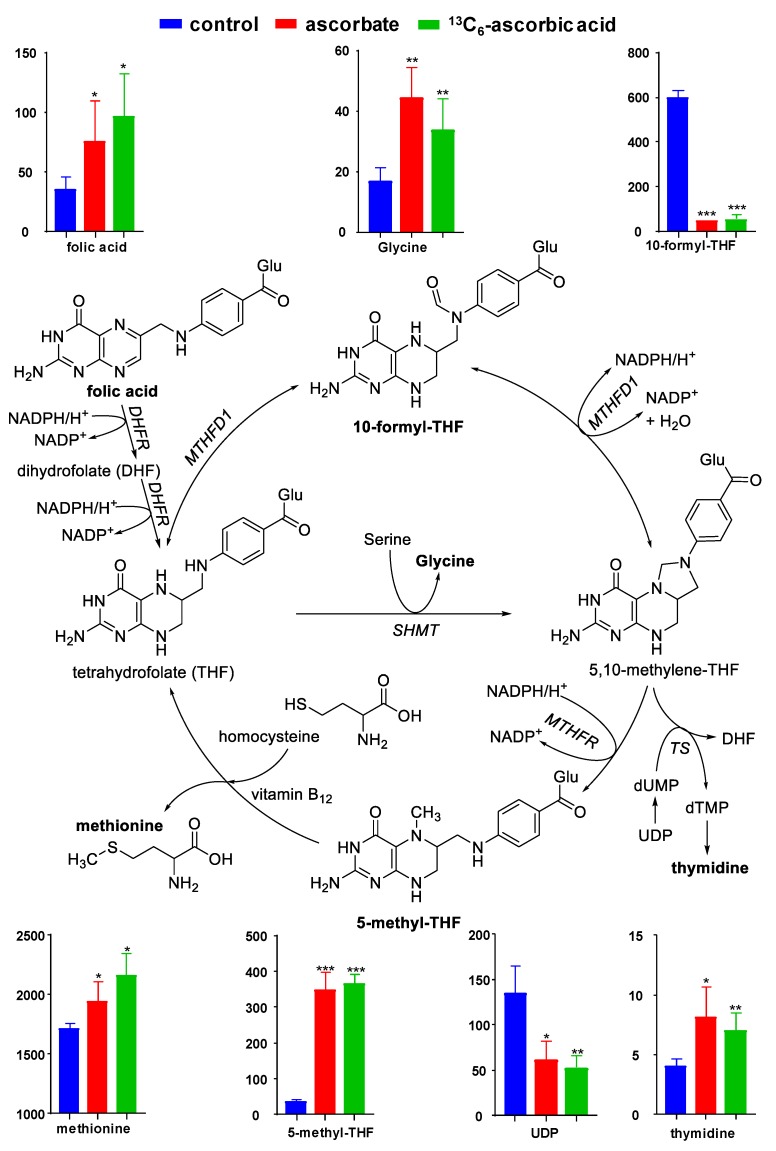
Simplified one-carbon cycle and associated pathways. Relative cellular levels of indicated metabolites are presented in the bar graphs (y-axes indicate peak areas expressed as 10^3^ counts per second). Enzyme abbreviations: DHFR, dihydrofolate reductase; Glu, glutamic acid; MTHFD1, 5,10-methylene-THF dehydrogenase 1; MTHFR, 5,10-methylene-THF reductase; SHMT, serine hydroxymethyl transferase; TS, thymidylate synthase. Asterisks (*) indicate statistical significance between the control and treatment (*: *p* ≤ 0.05, **: *p* ≤ 0.01, ***: *p* ≤ 0.001).

One-way ANOVA of the top 50 differentiating metabolites (Table 1) revealed that the relative cellular levels of folic acid, 5-methyl-THF, 10-formyl-THF, methionine sulfoxide, UDP, glycine, and thymidine changed significantly in response to AA-treatment (*n* = 3/group, *p* < 0.05, Figure 2 and supplementary Table S1). Although homocysteine was below the limit of integration, its oxidized form (homocysteic acid) was quantifiable but the change did not reach statistical significance (FC 0.69, *p* = 0.08; AA/Control, supplementary Table S1). These metabolites are either part of the folate-mediated one-carbon cycle or associated pathways (Figure 3). Their change in relative cellular levels in response to AA-treatment, shown in bar graphs, indicates that AA facilitates the reductive conversion of 10-formyl-THF into 5-methyl-THF. 

## 4. Discussion

The most significant changes in our untargeted metabolomics data as the result of exposure to AA were related to the folate-dependent one-carbon cycle and associated pathways that branch off this cycle (Figure 3). The observed AA-related decrease in 10-formyl-THF and concomitant increase in the cellular levels of 5-methyl-THF indicates that AA increases the activity of the folate-dependent one-carbon cycle. Stokes made a similar observation in a scurvy patient whose major urinary folate was 10-formyl-THF before AA treatment and 5-methyl-THF after AA therapy, concluding that AA “keeps the folate metabolism pool in action” [19]. Although we did not detect 5,10-methylene-THF, the intermediate in the conversion of 10-formyl-THF into 5-methyl-THF, we did observe an AA-related increase in the 5,10-methylene-THF-dependent formation of thymidine (a dTMP metabolite) and decrease in UDP. In DNA synthesis, UDP forms dUMP, which is the substrate of thymidylate synthase to form dTMP. THF can be converted directly into 5-methyl-THF by the enzyme serine hydroxymethyl transferase (SHMT, Figure 3), which consumes serine and produces glycine [20]. Although we observed a significant increase in the levels of glycine following AA treatment with no change in serine levels, we cannot draw conclusions regarding this pathway because both amino acids are components of the DMEM growth medium and because both amino acids are non-essential and can be formed and consumed in a multitude of other pathways. Regarding 5-methyl-THF as a co-substrate for the methionine synthase-mediated conversion of homocysteine into methionine, we observed an increase in the levels of methionine (Figure 3). This increase in methionine reflects its regeneration from homocysteine because the total amount of methionine, an essential amino acid, was provided by the growth medium. Taken together, the AA-induced changes in the metabolome of C2C12 cells appear to be a reflection of AA facilitating the folate-dependent one-carbon cycle and associated biochemical pathways (Figure 3). As the steps involved in the reductive conversion of 10-formyl-THF into 5-methyl-THF via 5,10-methylene-THF, are catalyzed by the NAD(P)H-dependent enzymes MTHFD and MTHFR, respectively, it is conceivable that AA plays a role in sparing NAD(P)H for reductive folate cycling. In scorbutic guinea pigs, for instance, cells can utilize a bioavailable form of glutathione (GSH) to delay the fall in tissue AA concentrations [21]. The authors conclude that AA and GSH “can spare each other” [21]. Because both AA and GSH require reducing equivalents from NADPH for the enzymatic recovery of their reduced forms in redox reactions [8], AA supplementation effectively spares NADPH by partially covering for GSH. The available NADPH can be used for a multitude of dehydrogenases involved in the detoxification ROS as well as their products [8]. We have previously observed that THP1 monocytes grown in the absence of AA showed greater cellular protein damage than AA-adequate cells due to AA’s ability to facilitate the detoxification of the lipid peroxidation product, 4-hydroxy-2(*E*)-nonenal [22], by enhancing the activities of the NADPH-dependent enzyme aldo-keto reductase and the NADH-dependent enzyme alcohol dehydrogenase [23]. AA can also directly scavenge the lipid peroxidation-derived acrolein by forming a Michael adduct [3] which would spare NADPH for other biochemical pathways, including the reductive part of the folate cycle. Conversely, Fan et al. [24] have shown that in proliferating HEK293T cells, grown in the absence of AA, the folate cycle turns counterclockwise to generate 10-formyl-THF and NADPH. This finding is in agreement with our results that in AA-deficient cells the levels of 10-formyl-THF are higher than in AA-adequate cells. We attribute the lack of change in cellular GSH and NADPH levels following AA supplementation to compensatory effects.

The distribution of folate metabolite pools in our study is in agreement with the principal folate metabolites found in foods, i.e., 10-formyl-THF and 5-formyl-THF [20]. However, food folates and intracellular folates of mammalian cells are primarily present in the form of polyglutamic acid derivatives that contain 3–9 Glu units conjugated with the carboxyl group of the *p*-aminobenzoic acid moiety of folate. In vivo, these polyglutamate units are hydrolyzed to mono-Glu forms which are recognized by specific folate receptors for cellular uptake [20]. We did not detect oligo- or poly-Glu forms of the folate metabolites in the lysates of C2C12 cells, which indicates that the form provided by the growth medium, i.e., the mono-Glu form of oxidized folic acid, is not converted into poly-Glu forms in C2C12 cells, which requires pteroylpolyglutamate synthetase (ligase) enzymes. By contrast, human fibroblasts and Chinese hamster ovary cells grown in culture accumulate polyglutamate folates [25,26]. The higher levels of oxidized monoglutamate folic acid in the AA-supplemented cells cannot be explained by higher ligase activity in the AA-deficient cells and probably reflects higher degradation of folates into pteridine and *p*-aminobenzoic acid products in the control cells.

10-Formyl-THF is a co-substrate for the de novo biosynthesis of purines as it delivers two carbon atoms of the purine ring structure [11]. Although we observed lower levels of 10-formyl-THF in the AA-treated cells consistent with its utilization for purine biosynthesis, the expected increase in levels of the purine, guanine, did not reach significance (1.3-fold, *p* = 0.055). There are multiple explanations for the metabolic changes because purine biosynthesis involves other substrates, including glutamine, glycine, and aspartate, and because there are multiple ways to interconvert purine forms such as their (deoxy)ribonucleoside and (deoxy)ribonucleotides [11]. We detected only one *N*-formyl derivative of THF which we identified as 10-formyl-THF by careful analysis of its MS/MS fragmentation pattern which unequivocally placed the formyl substituent on the nitrogen atom of the *p*-aminobenzoic acid moiety of THF, i.e., position 10 in THF. We excluded the possibility that the *N*-formyl form was 5-formyl-THF based on chromatographic and mass spectral comparison with an authentic sample of 5-formyl-THF. The detection of only one *N*-formyl regioisomer is somewhat unexpected because both forms can be reversibly interconverted by isomerization [11] and thus both would be detected assuming similar detection limits. 

Despite the increase in the relative cellular levels of methionine following AA exposure, we observed an AA-related decrease in methionine sulfoxide. Methionine is highly sensitive to oxidation by reactive oxygen species (ROS) and thus the levels of methionine sulfoxide can be considered a cellular biomarker of oxidative stress. The lower levels of methionine sulfoxide in the AA-treated cells compared to the control cells reflects the cellular antioxidant activity of AA (Figure S3). This effect can be explained by AA’s ability to scavenge ROS, thus preventing oxidation of methionine, or by AA facilitating the enzymatic recovery of methionine from methionine sulfoxide, which is catalyzed by methionine sulfoxide reductase (Msr) in a thioredoxin (Trx)- and NADPH-dependent manner [8,27,28]. As thioredoxin is integral to preserving an adequate redox status of cells as well, for instance by recovering reduced glutathione (GSH) from oxidized glutathione (GSSG) [29], AA indirectly assists Trx in its antioxidant activity by scavenging ROS thereby sparing NADPH needed for the regeneration of Trx from its oxidized form.

Elevated plasma levels of homocysteine (Hcy) are associated with cardiovascular disease while a causal relationship has not been established and the underlying mechanism(s) remain(s) poorly understood. Several clinical studies have tested the hypothesis that lowering Hcy by nutritional or pharmacological means reduces cardiovascular risk [30]. One such approach is folate therapy (with or without co-supplementation with vitamins B6 and B12) aimed at facilitating the conversion of Hcy into methionine by increasing the activity of the 5-methyl-THF-dependent enzyme, methionine synthase. A meta-analysis of several such trials revealed an average 25% reduction in plasma Hcy levels but the folate supplementation had no significant effect on major vascular events [30]. On the other hand, the China Stroke Primary Prevention Trial with 20,702 individuals reported a significant beneficial effect of folate supplementation in primary stroke prevention [31]. Unsatisfactory evidence-based support for the folate/Hcy hypothesis has led to the notion that perhaps Hcy is not the root cause of cardiovascular disease but that it creates or reflects a prooxidant environment characterized by decreased bioavailability of nitric oxide which negatively affects vascular function. Others have suggested that prolonged exposure to high plasma Hcy levels induces proatherogenic epigenetic alterations that persist long after subsidence of plasma Hcy [32]. Therefore, low-cost therapies that lower Hcy levels may be attractive for patients with hyperhomocysteinemia. 

In an observational study with 20 men and 40 women older than 50 years, serum Hcy correlated negatively with circulating levels of ascorbate (*r* = −0.30, *p* < 0.05) and folate (*r* = −0.31, *p* < 0.05) [33]. AA supplementation (500 mg/day) resulted in a significant 40% increase in erythrocyte folate in a study population comprising 65 male and 35 female Italian smokers without lowering Hcy levels [10]. High-dose AA supplementation (4.5 g/day) in 44 patients with coronary heart disease and without clinical ascorbate deficiency did not change plasma Hcy levels despite a marked increase in plasma ascorbate [34]. Our present cell culture study suggests that co-supplementation with folic acid and AA may activate the folate-mediated one-carbon cycle thereby promoting the conversion of Hcy into methionine while reducing cellular oxidative stress. ClinicalTrials.gov lists some 20 studies aimed at lowering Hcy with folic acid, combinations of B vitamins, prescription drugs, or with vitamin-prescription drug combinations, but none of the studies include(d) co-supplementation with AA. [35]. Acknowledging the limitation that the experiments were performed with one non-human-derived cell line, we propose a mechanism-based rationale for co-supplementation with folic acid and AA in humans, especially in elderly patients diagnosed with hyperhomocysteinemia and who are at risk for developing inadequate vitamin C status [2].

## 5. Conclusions

Our experiments with C2C12 myoblasts grown in the presence and absence of AA suggest that AA activates the folate-mediated one-carbon cycle thereby promoting the 5,10-methylene-THF-dependent formation of thymidine and facilitating the 5-methyl-THF-dependent conversion of Hcy into methionine. From a mechanistic perspective, AA appears to spare NAD(P)H and so increase availability of reducing equivalents for the reductive steps from 10-formyl-THF to 5-methyl-THF via 5,10-methylene-THF. The findings are relevant to nutritional or pharmacological treatment of hyperhomocysteinemia because the activity of the folate cycle appears to depend on AA concentration. Furthermore, our mechanistic findings provide a rationale for exploring the benefits of low-cost co-supplementation with AA in folate therapy of hyperhomocysteinemia.

## Figures and Tables

**Table 1:** Top 50 annotated compounds (one-way ANOVA followed by Tukey’s HSD (honestly significant difference) post-hoc analysis and Holm FDR (false discovery rate) correction; FC > 1.2, *p* ≤ 0.005) sorted by the significance of *p*-value for the comparison AA/Control. The complete list for the 204 annotated compounds in alphabetical order is presented in Table S2.

Compound	*m/z*	Retention Time (min)	Formula	Error ^1^	FC ^2^	*p*-Value
5-methyl-THF	460.1938	14.28	C_2_0H_25_N_7_O_6_	0.26	9.97	8.07 × 10^−9^
10-formyl-THF	474.1731	16.96	C_2_0H_23_N_7_O_7_	0.05	0.08	6.31 × 10^−7^
Pantothenic acid	218.1035	10.51	C_9_H_17_NO_5_	1.08	1.60	8.35 × 10^−7^
Guanidinoacetate	118.0603	4.74	C_3_H_7_N_3_O_2_	4.32	2.67	4.17 × 10^−6^
Methionine sulfoxide	166.0526	4.99	C_5_H_11_NO_3_S	4.08	0.71	4.90 × 10^−5^
Phosphodimethylethanolamine	192.0320	5.10	C_4_H_12_NO_4_P	0.92	1.95	7.95 × 10^−5^
PE(22:1/14:0)	746.5671	26.99	C_41_H_8_0NO_8_P	1.19	1.57	1.91 × 10^−4^
Urate	169.0351	5.97	C_5_H_4_N_4_O_3_	0.63	2.69	2.63 × 10^−4^
LysoPC(18:1)	522.3545	25.88	C_26_H_52_NO_7_P	0.78	1.47	4.31 × 10^−4^
Phenylacetylglycine	192.0665	17.88	C_1_0H_11_NO_3_	0.59	1.66	7.95 × 10^−4^
PE(18:1/18:0)	744.5530	26.96	C_41_H_8_0NO_8_P	0.11	1.77	8.59 × 10^−4^
Adenosine-5’-diphosphoglucose	428.0365	4.87	C_1_0H_15_N_5_O_1_0P_2_	0.44	0.60	9.18 × 10^−4^
6-keto-PGF1	393.2249	22.40	C_2_0H_34_O_6_	0.34	3.37	9.93 × 10^−4^
PE(22:1/14:1)	744.5529	26.99	C_41_H_78_NO_8_P	0.33	1.43	1.22 × 10^−3^
D-glucose 6-phosphate	259.0225	4.25	C_6_H_13_O_9_P	0.16	0.38	1.31 × 10^−3^
Adenosine 5’-monophosphate	346.0558	5.41	C_1_0H_14_N_5_O_7_P	0.15	0.57	1.61 × 10^−3^
Glycerate	105.0188	5.03	C_3_H_6_O_4_	2.27	4.38	1.65 × 10^−3^
Uridine diphosphate (UDP)	405.0094	4.07	C_9_H_14_N_2_O_12_P_2_	1.36	0.42	1.73 × 10^−3^
Thymidine	241.0831	11.03	C_1_0H_14_N_2_O_5_	3.33	1.90	1.81 × 10^−3^
Glycocholate	464.3009	23.90	C_26_H_43_NO_6_	6.89	1.81	1.85 × 10^−3^
LysoPG(16:0)	507.2694	24.33	C_22_H_45_O_9_P	0.42	1.38	1.96 × 10^−3^
LysoPE(18:1)	478.2934	24.90	C_23_H_46_NO_7_P	2.54	1.39	1.97 × 10^−3^
Glycerol 2-phosphate	171.0060	4.44	C_3_H_9_O_6_P	0.25	1.28	2.08 × 10^−3^
Hippurate	180.0650	17.08	C_9_H_9_NO_3_	1.02	1.46	2.13 × 10^−3^
Adenosine 5’-diphosphate	426.0221	4.85	C_1_0H_15_N_5_O_1_0P_2_	0.21	0.60	2.38 × 10^−3^
LysoPC(16:1)	538.3144	25.28	C_24_H_48_NO_7_P	1.77	1.35	2.46 × 10^−3^
Glycine	76.0385	4.79	C_2_H_5_NO_2_	1.84	2.31	2.86 × 10^−3^
5’-CMP	324.0591	4.77	C_9_H_14_N_3_O_8_P	0.03	0.65	2.97 × 10^−3^
Adenosine 3’,5’-diphosphate	450.0186	4.97	C_1_0H_15_N_5_O_1_0P_2_	0.20	0.58	4.39 × 10^−3^
NAD	662.1024	6.27	C_21_H_27_N_7_O_14_P_2_	3.00	0.50	4.51 × 10^−3^
Guanosine 5’-monophosphate	362.0507	5.26	C_1_0H_14_N_5_O_8_P	2.98	0.60	4.63 × 10^−3^
Aminoadipic acid	162.0755	4.92	C_6_H_11_NO_4_	4.20	0.78	4.70 × 10^−3^
N-acetyl-D-galactosamine	220.0826	5.16	C_8_H_15_NO_6_	1.29	0.46	4.80 × 10^−3^
Deoxyguanosine 5’-monophosphate	346.0556	6.99	C_1_0H_14_N_5_O_7_P	0.55	0.36	5.54 × 10^−3^
PE(16:0/18:1)	716.5231	26.78	C_39_H_76_NO_8_P	0.62	1.54	6.36 × 10^−3^
PS(16:0/16:1)	732.4822	26.53	C_38_H_72_NO_1_0P	0.07	0.67	7.14 × 10^−3^
PE(18:0/18:1)	766.5385	27.08	C_41_H_8_0NO_8_P	1.01	1.38	8.03 × 10^−3^
Succinate	117.0188	6.72	C_4_H_6_O_4_	2.85	0.64	8.12 × 10^−3^
CMP	322.0447	4.74	C_9_H_14_N_3_O_8_P	0.49	0.66	8.79 × 10^−3^
Glycerylphosphorylethanolamine	214.0486	4.49	C_5_H_14_NO_6_P	11.19	0.75	1.19 × 10^−2^
Phosphocreatine	212.0427	4.97	C_4_H_1_0N_3_O_5_P	3.82	0.69	1.19 × 10^−2^
PE-NMe(22:5/18:1)	850.5601	29.08	C_46_H_8_0NO_8_P	0.10	1.50	1.29 × 10^−2^
D-ribose 5-phosphate	229.0119	4.46	C_5_H_11_O_8_P	0.08	0.44	1.29 × 10^−2^
Indolelactic acid	204.0666	18.62	C_11_H_11_NO_3_	0.13	1.33	1.31 × 10^−2^
Pyridoxine	170.0806	6.74	C_8_H_11_NO_3_	0.66	1.29	1.38 × 10^−2^
Cytidine diphosphate choline	489.1149	5.33	C_14_H_26_N_4_O_11_P_2_	0.64	0.68	1.40 × 10^−2^
N-acetyl-D-glucosamine	222.0968	5.17	C_8_H_15_NO_6_	0.50	0.80	1.42 × 10^−2^
Cytidine 2’,3’-cyclic monophosphate	306.0486	5.17	C_9_H_12_N_3_O_7_P	0.14	1.37	1.44 × 10^−2^
Folic acid	442.1468	17.85	C_19_H_19_N_7_O_6_	0.34	2.45	1.45 × 10^−2^
PE(15:0/24:1)	832.6058	28.87	C_44_H_86_NO_8_P	0.73	1.34	1.52 × 10^−2^

Abbreviations: PE—phosphatidylethanolamine, PG—phosphatidylglycerol, and PS—phosphatidylserine. **^1^** Error in ppm. **^2^** Fold change AA/control.

## Supplementary Materials

The following are available online at https://www.mdpi.com/2076-3921/9/3/217/s1, **Figure S1:** Principal component analysis of the metabolomes of sham- and AA-treated C2C12 cells. Each set of dots represents a sample analyzed by LC–MS/MS. QC samples were analyzed every fifth LC run. Metabolites with CV > 25% were excluded from the analysis. **Figure S2:** Extracted ion chromatograms showing the total ion currents of the [M – H]^−^ ion of AA. A representative sample from each experimental group is displayed. The *m/z* 175.028 [M – H]^−^ ion of AA was absent in the control sample (blue) and present only in the sodium ascorbate treatment group (pink). The *m/z* 181.045 [M – H]^−^ ion corresponding to ^13^C_6_-ascorbic acid was only observed in samples treated with this AA isotopologue. MS/MS spectra recorded for the two AA isotopologues are displayed in the panels on the right. **Figure S3. (a)** Relative levels of methionine and methionine sulfoxide (Met-SO), and **(b)** Relative levels of Met-SO scaled to 100%. Panel **(a)** shows that the AA-related increase in methionine does not account for the decrease in Met-SO in the AA-treated cells. Asterisks (*) indicate statistical significance between the control and treatment (*: *p* ≤ 0.05, ***: *p* ≤ 0.001). **Table S1**: Compounds with CV < 25% on QC samples injected every five LC runs. Level 1 annotations were obtained from an in-house compound library consisting of >700 authentic IROA standards (IROA Technology, Bolton, MA). Level 2 annotations were obtained by Progenesis QI^TM^ software with Metlin^TM^ plugin V1.0.6499.51447 (NonLinear Dynamics, United Kingdom) using experimental MS/MS fragmentation, and Human Metabolome Database (HMDB) using in silico MS/MS fragmentation. Adduct declustering was implemented by using the Progenesis deconvolution algorithm. **Table. S2.** One-way analysis of variance followed by Tukey’s HSD post-hoc analysis and Holm FDR correction. Annotated compounds sorted by alphabetical order. Fold change and *p*-value are presented for the comparison of AA/Control.
