# Supplementary material for: Vitamin C Activates the Folate-Mediated One-Carbon Cycle in C2C12 Myoblasts
Source: Antioxidants (Basel). 2020 Mar 5;9(3):217. doi: 10.3390/antiox9030217 (PMC7139526; doi:10.3390/antiox9030217)
Supplement: Supplementary file 1 [file antioxidants-09-00217-s001.pdf]

antioxidants

MDPI

Supplementary Materials

Vitamin C activates the folate-mediated one-carbon cycle in C2C12 myocytes

Armando Alcazar Magana, Ralph L. Reed, Rony Koluda, Cristobal L. Miranda, Claudia S. Maier, Jan F. Stevens*

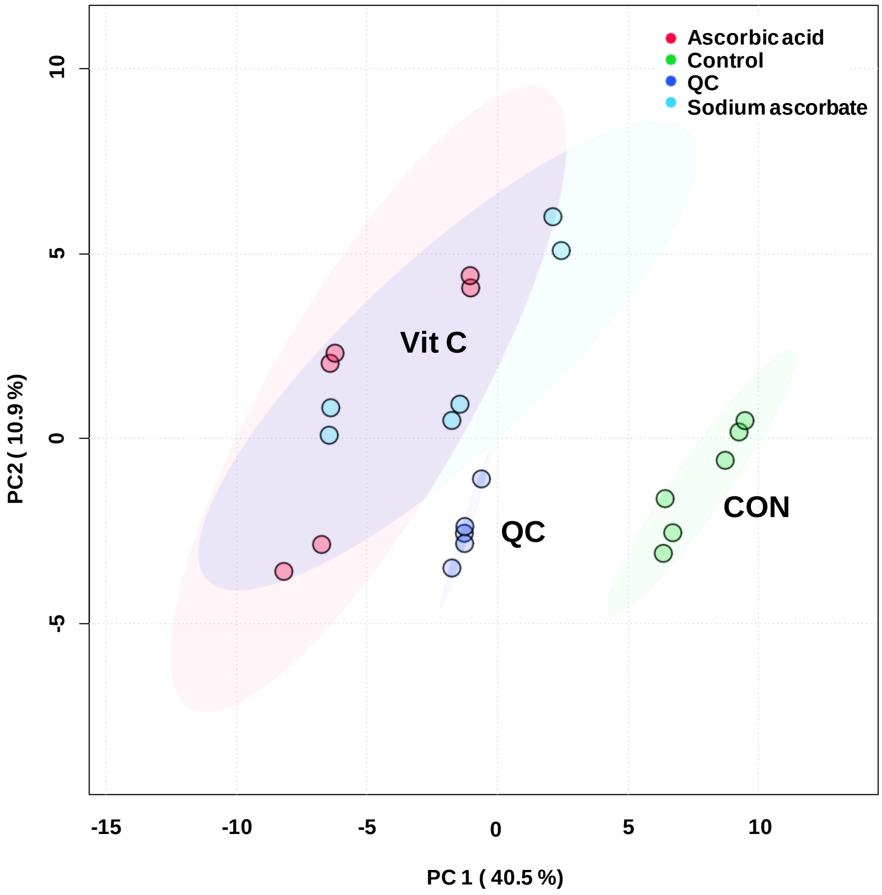

Figure S1. Principal component analysis of the metabolomes of sham- and AA-treated C2C12 cells. Each set of dots represents a sample analyzed by LC-MS/MS. QC samples were analyzed every fifth LC-run. Metabolites with CV > 25% were excluded from the analysis.

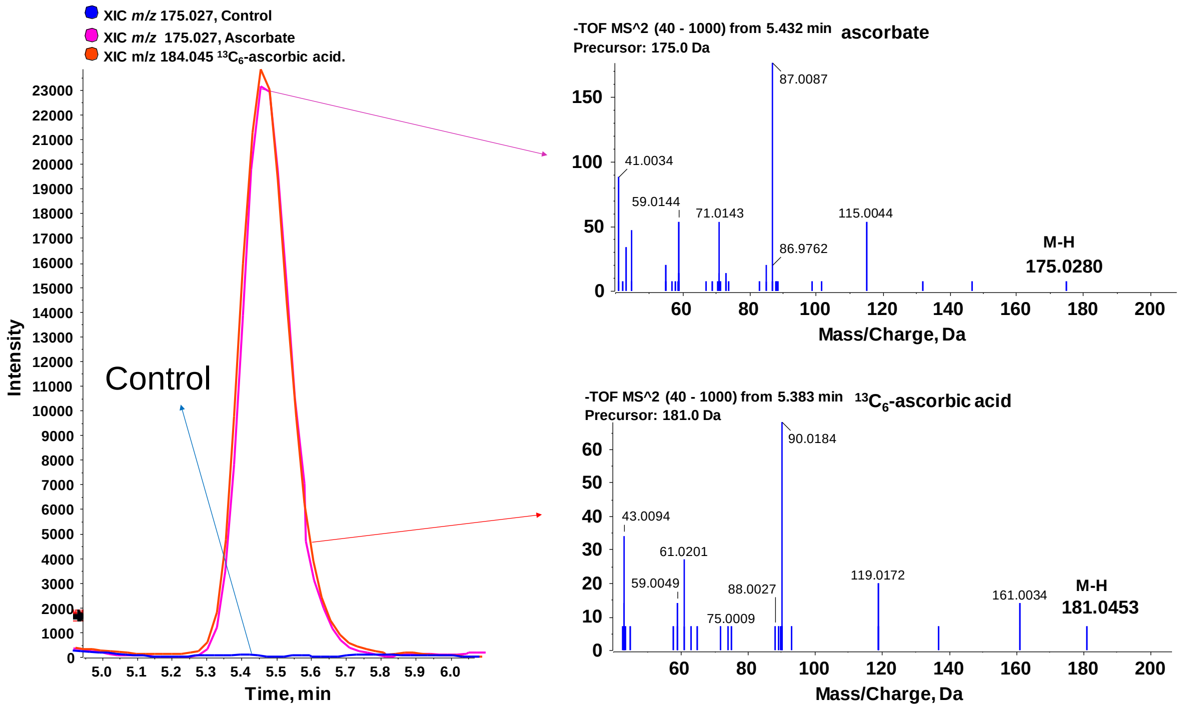

Figure S2. Extracted ion chromatograms showing the total ion currents of the [M-H]⁻ ion of AA. A representative sample from each experimental group is displayed. The *m/z* 175.028 [M-H]⁻ ion of AA was absent in the control sample (blue) and present only in the sodium ascorbate treatment group (pink). The *m/z* 181.045 [M-H]⁻ ion corresponding to $^{13}C_6$-ascorbic acid was only observed in samples treated with this AA isotopologue. MS/MS spectra recorded for the two AA isotopologues are displayed in the panels on the right.

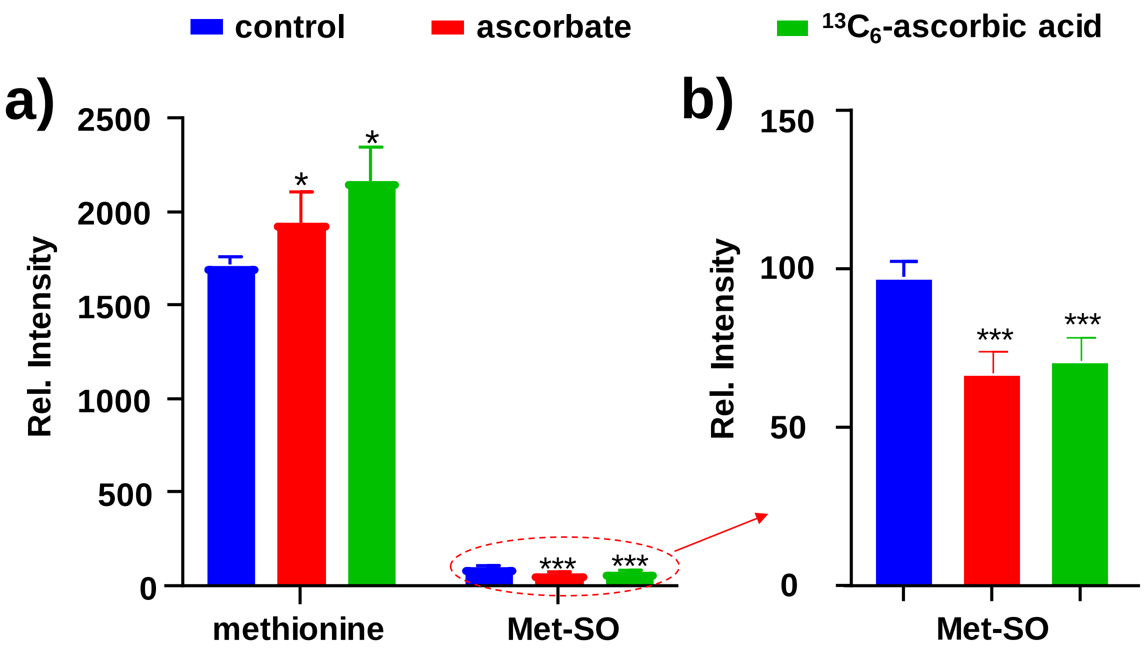

Figure S3. (a) Relative levels of methionine and methionine sulfoxide (Met-SO), and **(b)** Relative levels of Met-SO scaled to 100%. Panel **(a)** shows that the AA-related increase of methionine does not account for the decrease of Met-SO in the AA-treated cells. Asterisks (*) indicates statistical significance between the control and treatment (*: $p \leq 0.05$, ***: $p \leq 0.001$).

antioxidants

MDPI

Table S1

Compounds with CV<25% on QC’s sample injected every five LC runs. Level 1 annotations were obtained from an in-house compound library consisting of >700 authentic IROA standards (IROA Technology, Bolton, MA). Level 2 annotations were obtained by Progenesis QI™ software with Metlin™ plugin V1.0.6499.51447 (NonLinear Dynamics, United Kingdom) using experimental MS/MS fragmentation, and Human Metabolome Database (HMDB) using *in silico* MS/MS fragmentation. Adduct declustering was implemented by using Progenesis deconvolution algorithm.

Accepted Description	*m/z*	RT (min)	ESI Mode	Detected Adducts	Formula	Error (ppm)
			**Level 1 annotations, In-house library**			
1,2-Dipalmitoyl-sn-glycerol	591.4951	27.17	POS	M+H$^+$-H$_2$O, M+NH$_4^+$, M+Na$^+$	$C_{35}H_{68}O_5$	1.21
12-Hydroxydodecanoic acid	239.1619	23.42	POS	M+Na$^+$	$C_{12}H_{24}O_3$	0.53
3(4-Hydroxyphenyl)lactate	181.0507	12.17	NEG	M-H$^+$	$C_9H_{10}O_4$	0.24
5'-CMP	324.0591	4.77	POS	M+H$^+$, M+Na$^+$	$C_9H_{14}N_3O_8P$	0.03
5-Methyl-THF	460.1938	14.28	POS	M+H$^+$	$C_{20}H_{25}N_7O_6$	0.26
5'-Methylthioadenosine	298.0969	16.03	POS	M+H$^+$, M+Na$^+$	$C_{11}H_{15}N_5O_3S$	0.08
Adenine	136.0612	9.14	POS	M+H$^+$	$C_5H_5N_5$	3.99
Adenosine	268.1039	9.11	POS	M+H$^+$, M+Na$^+$	$C_{10}H_{13}N_5O_4$	0.39
Adenosine 3',5'-diphosphate	450.0186	4.97	POS	M+Na$^+$	$C_{10}H_{15}N_5O_{10}P_2$	0.20
Adenosine 5'-diphosphate	426.0221	4.85	NEG	M-H$^+$	$C_{10}H_{15}N_5O_{10}P_2$	0.21
Adenosine 5'-monophosphate	346.0558	5.41	NEG	M-H$^+$	$C_{10}H_{14}N_5O_7P$	0.15
Adenosine-5'-diphosphoglucose	428.0365	4.87	POS	M+H$^+$	$C_{10}H_{15}N_5O_{10}P_2$	0.44

Alanine	88.0398	4.51	NEG	$M-H^+$	$C_3H_7NO_2$	7.10
Allantoin	157.0365	5.03	NEG	$M-H^+$	$C_4H_6N_4O_3$	1.34
Aminocyclopropane carboxylic acid	102.0541	5.10	POS	$M+H^+$	$C_4H_7NO_2$	8.84
Arginine	175.1186	4.38	POS	$M+H^+$	$C_6H_{14}N_4O_2$	1.95
Asparagine	131.0457	4.46	NEG	$M-H^+$	$C_4H_8N_2O_3$	3.54
Aspartate	132.0297	4.49	NEG	$M-H^+$	$C_4H_7NO_4$	4.06
Azelaic acid	169.0869	20.46	NEG	$M-H_2O-H^+$	$C_9H_{16}O_4$	0.56
Betaine	118.0854	6.53	POS	$M+H^+$, $M+Na^+$	$C_5H_{11}NO_2$	7.47
Beta-nicotinamide mononucleotide	335.0642	5.38	POS	$M+H^+$	$C_{11}H_{15}N_2O_8P$	0.87
Carnitine	162.1119	6.05	POS	$M+H^+$	$C_7H_{15}NO_3$	3.38
Choline	104.1064	6.27	POS	$M+H^+$	$C_5H_{13}NO$	6.20
Citrate	215.0160	5.78	POS	$M+Na^+$	$C_6H_8O_7$	1.32
Citrulline	174.0882	4.79	NEG	$M-H^+$	$C_6H_{13}N_3O_3$	0.96
CMP	322.0447	4.74	NEG	$M-H^+$	$C_9H_{14}N_3O_8P$	0.49
Cytidine	244.0924	5.20	POS	$M+H^+$, $M+Na^+$	$C_9H_{13}N_3O_5$	1.68
Cytidine 2',3'-cyclic monophosphate	306.0486	5.17	POS	$M+H^+$, $M+Na^+$	$C_9H_{12}N_3O_7P$	0.14
Cytosine	112.0497	5.20	POS	$M+H^+$	$C_4H_5N_3O$	7.31
Deoxycytidine	272.0891	5.81	NEG	M+FA*-H	$C_9H_{13}N_3O_4$	1.07
Deoxyguanosine 5'-monophosphate	346.0556	6.99	NEG	$M-H^+$	$C_{10}H_{14}N_5O_7P$	0.55
Deoxyuridine	249.0495	8.45	NEG	$M+Na^+-2H$	$C_9H_{12}N_2O_5$	0.85
D-Glucose 6-phosphate	259.0225	4.25	NEG	$M-H^+$	$C_6H_{13}O_9P$	0.16
Dihydroorotate	141.0302	5.97	POS	$M+H^+-H_2O$	$C_5H_6N_2O_4$	4.86
D-Ribose 5-phosphate	229.0119	4.46	NEG	$M-H^+$	$C_5H_{11}O_8P$	0.08

Elaidic acid	303.2332	25.88	NEG	$M+Na^+-2H$	$C_{18}H_{34}O_2$	9.44
Ethanolamine phosphate	140.0112	4.44	NEG	$M-H^+$, $M+Na^+-2H$	$C_2H_8NO_4P$	4.54
Flavin adenine dinucleotide	784.1508	16.18	NEG	$M-H^+$, $M+Na^+-2H$	$C_{27}H_{33}N_9O_{15}P_2$	1.14
Folic acid	442.1468	17.85	POS	$M+H^+$	$C_{19}H_{19}N_7O_6$	0.34
Formylmethionine	176.0384	13.33	NEG	$M-H^+$	$C_6H_{11}NO_3S$	1.81
Fumarate	115.0031	6.39	NEG	$M-H^+$	$C_4H_4O_4$	4.93
Galactitol	183.0859	4.77	POS	$M+H^+-H_2O$, $M+H^+$	$C_6H_{14}O_6$	8.73
Gluconic acid	217.0300	4.67	NEG	$M-H_2O-H^+$, $M-H^+$, $M+Na^+-2H$	$C_6H_{12}O_7$	0.38
Glutamic acid	146.0456	4.64	NEG	$M-H_2O-H^+$, $M-H^+$	$C_5H_9NO_4$	4.20
Glutamine	145.0616	4.59	NEG	$M-H_2O-H^+$, $M-H^+$	$C_5H_{1}0N_2O_3$	3.36
Glutathione	306.0767	5.81	NEG	$M-H_2O-H^+$, $M-H^+$, $M+Na^+-2H$	$C_{1}0H_{17}N_3O_6S$	0.50
Glycerate	105.0188	5.03	NEG	$M-H^+$	$C_3H_6O_4$	5.08
Glycerol 2-phosphate	171.0060	4.44	NEG	$M-H^+$	$C_3H_9O_6P$	2.27
Glycine	76.0385	4.79	POS	$M+H^+$	$C_2H_5NO_2$	11.19
Glycocholate	464.3009	23.90	NEG	$M-H^+$	$C_{26}H_{43}NO_6$	1.84
Guanidinoacetate	118.0603	4.74	POS	$M+H^+$	$C_3H_7N_3O_2$	6.89
Guanosine 5'-monophosphate	362.0507	5.26	NEG	$M-H^+$	$C_{1}0H_{14}N_5O_8P$	0.02
Hippurate	180.0650	17.08	POS	$M+H^+$	$C_9H_9NO_3$	2.98
Inosine	291.0700	7.81	POS	$M+Na^+$	$C_{1}0H_{12}N_4O_5$	0.13
Isoleucine	132.1013	6.63	POS	$M+H^+$, $M+Na^+$	$C_6H_{13}NO_2$	4.54
Lactate	89.0240	5.99	NEG	$M-H^+$	$C_3H_6O_3$	4.33
L-aminocyclopropane-1-carboxylate	84.0436	4.64	POS	$M+H^+-H_2O$, $M+H^+$	$C_4H_7NO_2$	7.46
Lauric acid	199.1702	24.54	NEG	$M-H^+$	$C_{12}H_{24}O_2$	0.58
L-Oleoyl-rac-glycerol	379.2818	25.53	POS	$M+Na^+$	$C_{21}H_{4}0O_4$	0.29
L-Phenylalanine	164.0717	8.48	NEG	$M-H^+$	$C_9H_{11}NO_2$	0.29
Lysine	169.0943	4.15	POS	$M+Na^+$	$C_6H_{14}N_2O_2$	2.97

Malate	133.0138	5.36	NEG	$M-H_2O-H^+$, $M-H^+$	$C_4H_6O_5$	4.53
Methionine	150.0577	6.12	POS	$M+H^+$	$C_5H_{11}NO_2S$	4.04
Methylglutaric acid	167.0319	12.31	NEG	$M+Na^+-2H$	$C_6H_1 0O_4$	4.38
Methylhistidine	170.0919	4.71	POS	$M+H^+$	$C_7H_{11}N_3O_2$	3.12
N-Acetyl-D-galactosamine	220.0826	5.16	NEG	$M-H^+$, M+FA-H	$C_8H_{15}NO_6$	0.18
N-Acetyl-D-glucosamine	222.0968	5.17	POS	$M+H^+-H_2O$, $M+H^+$	$C_8H_{15}NO_6$	1.29
N-Acetyl-DL-methionine	214.0509	14.57	POS	$M+Na^+$	$C_7H_{13}NO_3S$	0.50
N-Acetyl-L-phenylalanine	206.0822	19.25	NEG	$M-H^+$	$C_{11}H_{13}NO_3$	0.15
N-acetylneuraminate	308.0991	4.85	NEG	$M-H^+$	$C_{11}H_{19}NO_9$	1.22
NADPH	726.0714	5.92	NEG	$M-H_2O-H^+$	$C_{21}H_3 0N_7O_{17}P_3$	2.46
Nicotinamide	123.0545	9.98	POS	$M+H^+$	$C_6H_6N_2O$	6.07
N-methyl-d-aspartic acid	180.0862	4.82	POS	$M+CH_3OH+H$	$C_5H_9NO_4$	3.00
Ophthalmic acid	290.1345	6.17	POS	$M+H^+$	$C_{11}H_{19}N_3O_6$	0.51
Palmitate	255.2332	25.54	NEG	$M-H^+$, M+FA-H	$C_{16}H_{32}O_2$	0.82
Palmitoleic acid	253.2174	25.41	NEG	$M-H^+$	$C_{16}H_3 0O_2$	0.50
Pantothenic acid	218.1035	10.51	NEG	$M-H^+$	$C_9H_{17}NO_5$	0.64
Phenylacetylglycine	192.0665	17.88	NEG	$M-H^+$	$C_1 0H_{11}NO_3$	0.49
Phenylalanine	164.0716	9.70	NEG	$M-H^+$	$C_9H_{11}NO_2$	0.59
Phosphocholine	184.0728	5.35	POS	$M+H^+$, $M+Na^+$	$C_5H_{14}NO_4P$	2.71
Phosphocreatine	212.0427	4.97	POS	$M+H^+$	$C_4H_1 0N_3O_5P$	1.74
Proline	116.0697	5.40	POS	$M+H^+$	$C_5H_9NO_2$	8.02
Pyridoxine	170.0806	6.74	POS	$M+H^+-H_2O$, $M+H^+$	$C_8H_{11}NO_3$	5.04
Rac-glycerol-l-myristate	325.2350	24.77	POS	$M+H^+-H_2O$, $M+H^+$, $M+Na^+$	$C_{17}H_{34}O_4$	0.30
Riboflavin	377.1456	19.05	POS	$M+H^+$, $M+Na^+$	$C_{17}H_2 0N_4O_6$	0.03
Serine	104.0344	4.46	NEG	$M-H^+$	$C_3H_7NO_3$	8.83
Spermine	203.2227	3.13	POS	$M+H^+$	$C_1 0H_{26}N_4$	1.55
Sphinganine	302.3055	23.19	POS	$M+H^+$	$C_{18}H_{39}NO_2$	0.53

Succinate	117.0188	6.72	NEG	$M-H^+$	$C_4H_6O_4$	4.16
Taurine	124.0070	4.51	NEG	$M-H^+$	$C_2H_7NO_3S$	2.85
Thiamine	265.1115	6.98	POS	$M+H^+$	$C_{12}H_{16}N_4OS$	0.96
Threonine	118.0505	4.64	NEG	$M-H^+$	$C_4H_9NO_3$	4.06
Thymidine	241.0831	11.03	NEG	$M-H^+$	$C_10H_{14}N_2O_5$	0.51
Thymidine 5'-monophosphate	323.0628	6.77	POS	$M+H^+$	$C_10H_{15}N_2O_8P$	3.33
Trimethyllysine	189.1593	5.20	POS	$M+H^+$	$C_9H_20N_2O_2$	2.34
Tryptophan	203.0827	11.97	NEG	$M-H^+$, $M+Na^+-2H$	$C_{11}H_{12}N_2O_2$	0.71
Tyrosine	204.0628	6.27	POS	$M+Na^+$	$C_9H_{11}NO_3$	1.93
Urate	169.0351	5.97	POS	$M+H^+$	$C_5H_4N_4O_3$	2.98
Uridine	245.0767	6.53	POS	$M+H^+$	$C_9H_{12}N_2O_6$	0.63
Uridine 5'-diphosphate	402.9948	4.98	NEG	$M-H^+$	$C_9H_{14}N_2O_{12}P_2$	0.23
Uridine-5-monophosphate	323.0290	4.84	NEG	$M-H^+$, $M+Na^+-2H$	$C_9H_{13}N_2O_9P$	1.36
Valine	116.0711	5.44	NEG	$M-H^+$	$C_5H_{11}NO_2$	5.12
**Level 2 annotations, experimental MS/MS fragmentation matching from METLIN™**						
10-Formyl-THF	474.1731	16.96	POS	$M+H^+$	$C_20H_{23}N_7O_7$	0.05
13-HpODE	311.2228	24.82	NEG	$M-H^+$	$C_{18}H_{32}O_4$	0.08
6-Keto-PGF1	393.2249	22.40	POS	$M+Na^+$	$C_20H_{34}O_6$	0.34
Acetylcarnitine	204.1227	6.77	POS	$M+H^+$	$C_9H_{17}NO_4$	1.68
Aminoadipic acid	162.0755	4.92	POS	$M+H^+-H_2O$, $M+H^+$	$C_6H_{11}NO_4$	4.20
Cytidine diphosphate choline (CDPcholine)	489.1149	5.33	POS	$M+H^+$, $M+Na^+$	$C_{14}H_{26}N_4O_{11}P_2$	0.64
D-Erythrose 4-phosphate	199.0015	5.08	NEG	$M-H^+$	$C_4H_9O_7P$	0.97
DG(18:0/18:0/0:0)	647.5577	27.45	POS	$M+H^+-H_2O$, $M+H^+$, $M+Na^+$	$C_{39}H_{76}O_5$	1.21
Glutamylglycine	203.0674	4.79	NEG	$M-H_2O-H^+$, $M-H^+$	$C_7H_{12}N_2O_5$	0.24
Glutathione, oxidized	613.1593	5.48	POS	$M+H^+$	$C_20H_{32}N_6O_{12}S_2$	0.17
Glycerophosphocholine	258.1100	5.45	POS	$M+H^+$	$C_8H_20NO_6P$	0.25

Guanine	152.0560	6.43	POS	$M+H^+$	$C_5H_5N_5O$	4.32
Hydroxyhexadecanoic acid	271.2277	24.92	NEG	$M-H^+$	$C_{16}H_{32}O_3$	0.47
Hypoxanthine	137.0457	6.74	POS	$M+H^+-H_2O$, $M+H^+$	$C_5H_4N_4O$	6.73
Indoleacrylic acid	188.0703	11.92	POS	$M+H^+$, $M+NH_4^+$	$C_{11}H_9NO_2$	1.85
Indolelactic acid	204.0666	18.62	NEG	$M-H^+$	$C_{11}H_{11}NO_3$	0.12
L-Tyrosine	180.0667	6.32	NEG	$M-H^+$	$C_9H_{11}NO_3$	0.49
LysoPC(18:1)	522.3545	25.88	POS	$M+H^+$	$C_{26}H_{52}NO_7P$	1.77
LysoPE(0:0/22:5)	528.3081	25.28	POS	$M+H^+$	$C_{27}H_{46}NO_7P$	0.78
LysoPE(18:1/0:0)	502.2877	24.90	POS	$M+H^+$, $M+Na^+$	$C_{23}H_{46}NO_7P$	1.37
Lysophosphatidylserine	526.3127	24.90	POS	$M+H^+-H_2O$, $M+H^+$, $M+Na^+$	$C_{24}H_{48}NO_9P$	2.36
LysoPI(18:0)	623.3158	24.70	POS	$M+H^+-H_2O$, $M+H^+$, $M+Na^+$	$C_{27}H_{53}O_{12}P$	1.23
Methionine sulfoxide	166.0526	4.99	POS	$M+H^+$, $M+Na^+$	$C_5H_{11}NO_3S$	4.08
MG(0:0/18:0/0:0)	381.2969	25.63	POS	$M+H^+-H_2O$, $M+H^+$, $M+NH_4^+$, $M+Na^+$	$C_{21}H_{42}O_4$	1.05
Monopalmitin	353.2661	25.26	POS	$M+H^+-H_2O$, $M+H^+$, $M+NH_4^+$, $M+Na^+$	$C_{19}H_{38}O_4$	0.02
Muramic acid	234.0977	4.69	POS	$M+H^+-H_2O$, $M+H^+$	$C_9H_{17}NO_7$	1.88
Nicotinamide adenine dinucleotide (NAD)	662.1024	6.27	NEG	$M-H^+$, $M+Na^+-2H$	$C_{21}H_{27}N_7O_{14}P_2$	0.78
PE(16:1/18:1)	716.5247	26.84	POS	$M+H^+$	$C_{39}H_{74}NO_8P$	3.12
PE(18:1/0:0)	478.2934	24.90	NEG	$M-H^+$, $M+Na^+-2H$	$C_{23}H_{46}NO_7P$	1.01
PG(16:0/0:0)	507.2694	24.33	POS	$M+H^+-H_2O$, $M+H^+$, $M+Na^+$	$C_{22}H_{45}O_9P$	0.19
PG(18:1/0:0)	533.2846	24.66	POS	$M+H^+-H_2O$, $M+H^+$, $M+Na^+$	$C_{24}H_{47}O_9P$	0.42
Phenyllactic acid	165.0555	17.94	NEG	$M-H^+$	$C_9H_10O_3$	1.42
Phosphodimethylethanolamine	192.0320	5.10	POS	$M+H^+$, $M+Na^+$	$C_4H_{12}NO_4P$	3.82
PS(18:1/0:0)	524.2974	24.70	POS	$M+H^+$, $M+Na^+$	$C_{24}H_{46}NO_9P$	1.68
PS(22:6/18:0)	836.5421	27.17	POS	$M+H^+$, $M+Na^+$	$C_{46}H_{78}NO_10P$	1.77
Pyridoxine phosphate	267.0587	6.58	POS	$M+H^+$, $M+NH_4^+$	$C_8H_{12}NO_6P$	0.66
Pyroglutamic acid	130.0491	6.77	POS	$M+H^+$, $M+Na^+$	$C_5H_7NO_3$	5.73

S-Adenosylhomocysteine	385.1289	6.74	POS	$M+H^{+}$	$C_{14}H_{2}0N_{6}O_{5}S$	0.21
S-Adenosylmethionine	399.1444	5.07	POS	$M+H^{+}$	$C_{15}H_{22}N_{6}O_{5}S$	0.17
Undecanedicarboxylic acid	243.1602	23.31	NEG	$M-H^{+}$	$C_{13}H_{24}O_{4}$	0.04
Uridine diphosphate (UDP)	405.0094	4.07	POS	$M+H^{+}$	$C_{9}H_{14}N_{2}O_{12}P_{2}$	0.27
**Level 2 annotations, In silico fragmentation matching from HMDB**						
5-Hydroxy-L-tryptophan	221.0915	6.93	POS	$M+H^{+}-H_{2}O$, $M+H^{+}$	$C_{11}H_{12}N_{2}O_{3}$	0.82
5-Methylcytidine	302.1010	5.46	NEG	M+FA-H	$C_{1}0H_{15}N_{3}O_{5}$	6.50
5-Tetradecenoic acid	271.1915	24.07	NEG	M+FA-H	$C_{14}H_{26}O_{2}$	0.00
Cer(18:1/24:1)	692.6189	27.18	NEG	M+FA-H	$C_{42}H_{81}NO_{3}$	1.41
Glycerylphosphorylethanolamine	214.0486	4.49	NEG	$M-H^{+}$	$C_{5}H_{14}NO_{6}P$	0.20
Homo-L-arginine	189.1344	4.51	POS	$M+H^{+}$	$C_{7}H_{16}N_{4}O_{2}$	1.02
L-Homocysteic acid	182.0129	6.34	NEG	$M-H^{+}$	$C_{4}H_{9}NO_{5}S$	0.17
LysoPC(16:0)	518.3214	25.51	POS	$M+Na^{+}$	$C_{24}H_{5}0NO_{7}P$	0.70
LysoPC(16:1)	538.3144	25.28	NEG	M+FA-H	$C_{24}H_{48}NO_{7}P$	1.36
N2-Fructopyranosylarginine	337.1718	4.46	POS	$M+H^{+}$	$C_{12}H_{24}N_{4}O_{7}$	0.17
PA(20:2/18:1)	771.5186	26.91	NEG	M+FA-H	$C_{41}H_{75}O_{8}P$	0.66
PC(14:0/20:1)	782.5662	28.73	POS	$M+Na^{+}$	$C_{42}H_{82}NO_{8}P$	1.08
PC(18:0/18:2)	830.5914	28.92	NEG	M+FA-H	$C_{44}H_{84}NO_{8}P$	0.32
PC(18:0/20:5)	852.5753	29.09	NEG	M+FA-H	$C_{46}H_{82}NO_{8}P$	0.89
PC(22:4/16:0)	838.5969	29.25	NEG	M+FA-H	$C_{46}H_{84}NO_{7}P$	0.16
PC(P-18:0/16:1)	788.5802	28.84	NEG	M+FA-H	$C_{42}H_{82}NO_{7}P$	1.26
PE(14:0/18:1)	690.5061	26.69	POS	$M+H^{+}$, $M+Na^{+}$	$C_{37}H_{72}NO_{8}P$	1.03
PE(14:0/22:1)	790.5605	28.50	NEG	M+FA-H	$C_{41}H_{8}0NO_{8}P$	0.15
PE(14:1/22:0)	768.5524	27.12	POS	$M+Na^{+}$	$C_{41}H_{8}0NO_{8}P$	1.43
PE(15:0/20:1)	776.5447	28.40	NEG	M+FA-H	$C_{4}0H_{78}NO_{8}P$	0.05
PE(15:0/20:2)	774.5287	28.40	NEG	M+FA-H	$C_{4}0H_{76}NO_{8}P$	0.43
PE(15:0/22:1)	804.5761	28.65	NEG	M+FA-H	$C_{42}H_{82}NO_{8}P$	0.10

PE(15:0/22:2)	802.5606	28.68	NEG	M+FA-H	$C_{42}H_{8}0NO_{8}P$	0.29
PE(15:0/24:1)	832.6058	28.87	NEG	M+FA-H	$C_{44}H_{86}NO_{8}P$	1.94
PE(16:0/18:1)	716.5231	26.78	NEG	$M-H^{+}$	$C_{39}H_{76}NO_{8}P$	0.73
PE(16:0/20:4)	738.5075	26.91	NEG	$M-H^{+}$	$C_{41}H_{74}NO_{8}P$	0.62
PE(18:0/18:1)	766.5385	27.08	NEG	$M+Na^{+}-2H$	$C_{41}H_{8}0NO_{8}P$	2.28
PE(18:1/18:0)	744.5530	26.96	NEG	$M-H^{+}$	$C_{41}H_{8}0NO_{8}P$	2.54
PE(18:2/16:0)	760.5133	26.70	NEG	M+FA-H	$C_{39}H_{74}NO_{8}P$	0.11
PE(18:2/16:1)	758.4971	26.68	NEG	M+FA-H	$C_{39}H_{72}NO_{8}P$	0.87
PE(20:2/16:0)	788.5437	26.86	NEG	M+FA-H	$C_{41}H_{78}NO_{8}P$	1.38
PE(20:2/16:1)	786.5285	26.83	NEG	M+FA-H	$C_{41}H_{76}NO_{8}P$	0.81
PE(20:4/P-16:0)	722.5122	27.01	NEG	$M-H^{+}$	$C_{41}H_{74}NO_{7}P$	1.07
PE(22:1/14:0)	746.5671	26.99	POS	$M+H^{+}$	$C_{41}H_{8}0NO_{8}P$	3.12
PE(22:1/14:1)	744.5529	26.99	POS	$M+H^{+}$	$C_{41}H_{78}NO_{8}P$	1.19
PE(22:6/16:0)	762.5082	27.06	NEG	$M-H^{+}$	$C_{43}H_{74}NO_{8}P$	0.33
PE(P-16:0/18:1)	700.5287	26.88	NEG	$M-H^{+}$	$C_{39}H_{76}NO_{7}P$	0.11
PE(P-16:0/20:5)	720.4969	27.01	NEG	$M-H^{+}$	$C_{41}H_{72}NO_{7}P$	0.61
PE(P-16:0e/0:0)	482.2922	23.98	NEG	M+FA-H	$C_{21}H_{44}NO_{6}P$	7.79
PE-NMe(18:0/18:3)	800.5441	28.68	NEG	M+FA-H	$C_{42}H_{78}NO_{8}P$	0.86
PE-NMe(20:0/20:2)	858.6197	29.13	NEG	M+FA-H	$C_{46}H_{88}NO_{8}P$	3.98
PE-NMe(20:0/22:6)	878.5911	29.31	NEG	M+FA-H	$C_{48}H_{84}NO_{8}P$	0.67
PE-NMe(20:1/22:6)	876.5762	29.35	NEG	M+FA-H	$C_{48}H_{82}NO_{8}P$	0.23
PE-NMe(22:4/16:0)	826.5601	28.79	NEG	M+FA-H	$C_{44}H_{8}0NO_{8}P$	0.34
PE-NMe(22:4/18:0)	854.5907	29.08	NEG	M+FA-H	$C_{46}H_{84}NO_{8}P$	1.21
PE-NMe(22:5/18:1)	850.5601	29.08	NEG	M+FA-H	$C_{46}H_{8}0NO_{8}P$	0.32
PE-NMe2(18:1/18:0)	818.5916	28.74	NEG	M+FA-H	$C_{43}H_{84}NO_{8}P$	0.10
PE-NMe2(20:2/16:0)	816.5765	28.77	NEG	M+FA-H	$C_{43}H_{82}NO_{8}P$	0.62
PI(18:0/20:4)	885.5490	27.34	NEG	$M-H^{+}$	$C_{47}H_{83}O_{13}P$	0.92

PI(18:2/22:2)	913.5810	27.70	NEG	M-H$^+$, M+FA-H	$C_{49}H_{87}O_{13}P$	0.13
PI(18:3/18:0)	905.5406	27.24	NEG	M+FA-H	$C_{45}H_{81}O_{13}P$	1.04
Prolyl-Hydroxyproline	229.1181	6.22	POS	M+H$^+$	$C_{1}0H_{16}N_{2}O_{4}$	0.82
PS(16:0/16:1)	732.4822	26.53	NEG	M-H$^+$, M+Na$^+$-2H	$C_{38}H_{72}NO_{1}0P$	0.08
PS(18:0/18:0)	812.5422	26.98	NEG	M+Na$^+$-2H	$C_{42}H_{82}NO_{1}0P$	0.07
PS(18:0/18:1)	810.5271	26.96	NEG	M+Na$^+$-2H	$C_{42}H_{8}0NO_{1}0P$	0.61
PS(20:4/18:1)	790.5021	27.03	NEG	M-H$_2$O-H$^+$	$C_{44}H_{76}NO_{1}0P$	0.93
PS(22:4/20:3)	842.5303	27.31	NEG	M-H$_2$O-H$^+$	$C_{48}H_{8}0NO_{1}0P$	4.41
PS(22:4/20:4)	840.5157	27.31	NEG	M-H$_2$O-H$^+$	$C_{48}H_{78}NO_{1}0P$	3.21
SM(d18:1/24:1)	857.6748	28.50	NEG	M+FA-H	$C_{47}H_{93}N_{2}O_{6}P$	0.66
TG(18:1/16:0/20:2)	907.7706	28.63	POS	M+Na$^+$	$C_{57}H_{1}0_{4}O_{6}$	2.20
TG(18:1/18:2/20:5)	903.7388	28.68	POS	M+H$^+$	$C_{59}H_{98}O_{6}$	5.29

* Abreviations: PA – phosphatidic acid, PE – phosphatidylethanolamine, PG – phosphatidylglycerol, PS – phosphatidylserine, DG – diglyceride, PC – phosphatidylcholine, PI – phosphatidylinositol, Cer – ceramides, SM – sphingomyelin, TG – triglyceride, DG – diglyceride, MG – monoglyceride

Table. S2. One-way analysis of variance followed by Tukey's HSD *post hoc* analysis and Holm FDR-correction. Annotated compounds are sorted by alphabetical order. Fold change and p-value are presented for the comparison of AA/Control.

Compound	FC[b]	p- value
1,2-Dipalmitoyl-sn-glycerol	0.91	1.28E-02
10-Formyl-THF	0.08	6.31E-07
12-Hydroxydodecanoic acid	1.03	2.02E-01
13-HpODE	1.04	4.86E-01
3(4-Hydroxyphenyl)lactate	0.94	3.40E-01
5'-CMP	0.65	2.97E-03
5-Hydroxy-L-tryptophan	0.89	4.71E-01
5-Methylcytidine	0.80	2.01E-02
5-Methyl-THF	9.97	8.07E-09
5'-Methylthioadenosine	1.40	6.86E-02
5-Tetradecenoic acid	0.87	9.03E-02
6-Keto-PGF1	3.37	9.93E-04
Acetylcarnitine	0.86	7.07E-02
Adenine	1.18	3.30E-01
Adenosine	1.28	2.15E-02
Adenosine 3',5'-diphosphate	0.58	4.39E-03
Adenosine 5'-diphosphate	0.60	2.38E-03
Adenosine 5'-monophosphate	0.57	1.61E-03
Adenosine-5'-diphosphoglucose	0.60	9.18E-04
Alanine	1.02	8.97E-01
Allantoin	1.19	1.74E-02
Aminoadipic acid	0.78	4.70E-03
Aminocyclopropane carboxylic acid	0.72	6.29E-02
Arginine	0.96	6.91E-01
Asparagine	0.97	6.89E-01
Aspartate	0.98	7.66E-01
Azelaic acid	1.45	3.77E-02
Betaine	1.06	4.14E-01
Beta-nicotinamide mononucleotide	1.06	6.77E-01
Carnitine	0.93	3.80E-01
Cer(18:1/24:1)	1.28	4.94E-02
Choline	1.13	1.89E-02
Citrate	1.12	1.88E-01
Citrulline	1.34	2.31E-01
CMP	0.66	8.79E-03

Cytidine	1.17	1.90E-02
Cytidine 2',3'-cyclic monophosphate	1.37	1.44E-02
Cytidine diphosphate choline	0.68	1.40E-02
Cytosine	1.11	2.45E-01
Deoxycytidine	0.72	2.48E-01
Deoxyguanosine 5'-monophosphate	0.36	5.54E-03
Deoxyuridine	0.92	1.91E-01
D-Erythrose 4-phosphate	1.09	4.55E-01
DG(18:0/18:0/0:0)	0.72	3.67E-02
D-Glucose 6-phosphate	0.38	1.31E-03
Dihydroorotate	0.99	9.02E-01
D-Ribose 5-phosphate	0.44	1.29E-02
Elaidic acid	2.02	1.45E-01
Ethanolamine phosphate	0.83	2.28E-01
Flavin adenine dinucleotide	0.69	1.94E-02
Folic acid	2.45	1.45E-02
Formylmethionine	1.12	7.42E-01
Fumarate	1.29	3.88E-02
Galactitol	1.15	6.46E-01
Gluconic acid	1.15	6.48E-03
Glutamic acid	0.86	2.93E-02
Glutamine	0.88	1.80E-03
Glutamylglycine	1.11	5.29E-01
Glutathione	0.72	8.18E-02
Glutathione, oxidized	1.00	8.11E-01
Glycerate	4.38	1.65E-03
Glycerol 2-phosphate	1.28	2.08E-03
Glycerophosphocholine	0.85	3.51E-02
Glycerylphosphorylethanolamine	0.75	1.19E-02
Glycine	2.31	2.86E-03
Glycocholate	1.81	1.85E-03
Guanidinoacetate	2.67	4.17E-06
Guanine	1.30	5.51E-02
Guanosine 5'-monophosphate	0.60	4.63E-03
Hippurate	1.46	2.13E-03
Homo-L-arginine	1.43	4.07E-02
Hydroxyhexadecanoic acid	0.86	1.64E-01
Hypoxanthine	1.09	3.90E-01
Indoleacrylic acid	1.35	1.36E-01
Indolelactic acid	1.33	1.31E-02
Inosine	1.06	5.90E-01
Isoleucine	1.07	4.37E-01

Lactate	0.96	4.17E-01
L-aminocyclopropane-1-carboxylate	0.85	4.47E-02
Lauric acid	0.82	4.05E-01
L-Homocysteic acid	0.69	8.07E-02
L-Oleoyl-rac-glycerol	0.96	7.56E-01
L-Phenylalanine	0.90	9.64E-02
L-Tyrosine	1.12	1.08E-02
Lysine	1.32	3.06E-02
LysoPC(16:0)	1.19	2.54E-02
LysoPC(16:1)	1.35	2.46E-03
LysoPC(18:1)	1.47	4.31E-04
LysoPE(0:0/22:5)	2.25	1.87E-02
LysoPE(18:1/0:0)	1.33	2.78E-02
Lysophosphatidylserine	0.92	5.82E-02
LysoPI(18:0/0:0)	1.15	1.06E-01
Malate	0.97	3.36E-01
Methionine	1.20	2.46E-02
Methionine sulfoxide	0.71	4.90E-5
Methylglutaric acid	1.23	6.66E-02
Methylhistidine	1.05	3.90E-01
MG(0:0/18:0/0:0)	1.02	8.29E-01
Monopalmitin	1.07	4.41E-01
Muramic acid	4.09	1.68E-01
N2-Fructopyranosylarginine	6.49	2.48E-02
N-Acetyl-D-galactosamine	0.46	4.80E-03
N-Acetyl-D-glucosamine	0.80	1.42E-02
N-Acetyl-DL-methionine	0.95	7.64E-01
N-Acetyl-L-phenylalanine	0.85	1.24E-01
N-acetylneuraminate	1.09	8.44E-01
NADPH	0.79	3.07E-01
Nicotinamide	1.06	6.70E-01
NAD	0.50	4.51E-03
N-methyl-d-aspartic acid	1.35	5.81E-02
Ophthalmic acid	1.11	2.63E-01
PA(20:2/18:1)	1.08	7.67E-01
Palmitate	0.90	4.54E-01
Palmitoleic acid	1.07	4.95E-01
Pantothenic acid	1.60	8.35E-07
PC(14:0/20:1)	1.33	2.49E-01
PC(18:0/18:2)	1.20	1.06E-01
PC(18:0/20:5)	1.15	3.61E-01
PC(22:4/16:0)	1.07	9.33E-01

PC(P-18:0/16:1)	1.25	4.22E-01
PE(14:0/18:1)	1.93	5.64E-02
PE(14:0/22:1)	1.08	6.94E-01
PE(14:1/22:0)	1.18	6.68E-03
PE(15:0/20:1)	1.04	7.98E-01
PE(15:0/20:2)	1.01	9.83E-01
PE(15:0/22:1)	1.13	4.38E-01
PE(15:0/22:2)	1.04	8.61E-01
PE(15:0/24:1)	1.34	1.52E-02
PE(16:0/18:1)	1.54	6.36E-03
PE(16:0/20:4)	1.35	7.32E-02
PE(16:1/18:1)	1.14	3.07E-01
PE(18:0/18:1)	1.38	8.03E-03
PE(18:1/0:0)	1.39	1.97E-03
PE(18:1/18:0)	1.77	8.59E-04
PE(18:2/16:0)	0.86	2.15E-02
PE(18:2/16:1)	0.82	1.84E-02
PE(20:2/16:0)	0.90	2.37E-01
PE(20:2/16:1)	0.90	1.58E-01
PE(20:4/P-16:0)	1.44	2.17E-02
PE(22:1/14:0)	1.57	1.91E-04
PE(22:1/14:1)	1.43	1.22E-03
PE(22:6/16:0)	1.40	1.55E-02
PE(P-16:0/18:1)	1.03	8.91E-01
PE(P-16:0/20:5)	1.30	1.42E-01
PE(P-16:0e/0:0)	1.15	2.50E-01
PE-NMe(18:0/18:3)	1.08	7.39E-01
PE-NMe(20:0/20:2)	1.43	1.36E-01
PE-NMe(20:0/22:6)	1.31	2.27E-01
PE-NMe(20:1/22:6)	1.08	5.64E-01
PE-NMe(22:4/16:0)	1.15	3.86E-01
PE-NMe(22:4/18:0)	1.23	2.27E-01
PE-NMe(22:5/18:1)	1.50	1.29E-02
PE-NMe2(18:1/18:0)	1.50	2.06E-02
PE-NMe2(20:2/16:0)	1.43	4.94E-02
PG(16:0/0:0)	1.38	1.96E-03
PG(18:1/0:0)	1.18	6.14E-02
Phenylacetylglycine	1.66	7.95E-04
Phenylalanine	0.97	5.81E-01
Phenyllactic acid	0.97	6.49E-01
Phosphocholine	0.99	7.78E-01
Phosphocreatine	0.69	1.19E-02

Phosphodimethylethanolamine	1.95	7.95E-05
PI(18:0/20:4)	0.83	6.37E-02
PI(18:2/22:2)	0.79	2.28E-02
PI(18:3/18:0)	0.91	5.89E-01
Proline	0.87	1.01E-02
Prolyl-Hydroxyproline	1.17	5.26E-02
PS(16:0/16:1)	0.67	7.14E-03
PS(18:0/18:0)	0.94	5.46E-01
PS(18:0/18:1)	0.92	3.49E-01
PS(18:1/0:0)	1.33	3.07E-02
PS(20:4/18:1)	1.18	1.18E-01
PS(22:4/20:3)	1.28	1.61E-01
PS(22:4/20:4)	1.55	3.41E-02
PS(22:6/18:0)	0.77	1.88E-02
Pyridoxine	1.29	1.38E-02
Pyridoxine phosphate	0.96	6.18E-01
Pyroglutamic acid	0.97	6.18E-01
Rac-glycerol-l-myristate	0.92	1.20E-01
Riboflavin	1.40	1.97E-02
S-Adenosylhomocysteine	1.09	2.00E-01
S-Adenosylmethionine	0.84	2.01E-01
Serine	0.98	9.43E-01
SM(d18:1/24:1)	0.90	5.06E-01
Spermine	3.36	2.11E-02
Sphinganine	0.95	5.50E-02
Succinate	0.64	8.12E-03
Taurine	0.85	6.55E-02
TG(18:1/16:0/20:2)	0.96	6.11E-01
TG(18:1/18:2/20:5)	0.92	8.17E-01
Thiamine	1.00	9.59E-01
Threonine	0.94	2.03E-01
Thymidine	1.90	1.81E-03
Thymidine 5'-monophosphate	1.10	7.77E-01
Trimethyllysine	1.16	3.46E-02
Tryptophan	1.08	5.31E-01
Tyrosine	1.16	2.14E-01
Undecanedicarboxylic acid	0.95	5.27E-01
Urate	2.69	2.63E-04
Uridine	0.93	5.44E-01
Uridine 5'-diphosphate	0.45	4.09E-02
Uridine diphosphate (UDP)	0.42	1.73E-03
Uridine-5-monophosphate	0.75	6.92E-02

Valine	1.23	1.44E-01

* Abreviations: PA – phosphatidic acid, PE – phosphatidylethanolamine, PG – phosphatidylglycerol, PS – phosphatidylserine, DG – diglyceride, PC – phosphatidylcholine, PI – phosphatidylinositol, Cer – ceramides, SM – sphingomyelin, TG – triglyceride, DG – diglyceride, MG – monoglyceride.
